# *Gpnmb*^*R*150*X *^allele must be present in bone marrow derived cells to mediate DBA/2J glaucoma

**DOI:** 10.1186/1471-2156-9-30

**Published:** 2008-04-10

**Authors:** Michael G Anderson, K Saidas Nair, Leslie A Amonoo, Adrienne Mehalow, Colleen M Trantow, Sharmila Masli, Simon WM John

**Affiliations:** 1 Department of Molecular Physiology and Biophysics, University of Iowa, Iowa City, Iowa USA; 2 Department of Ophthalmology and Visual Sciences, University of Iowa, Iowa City, Iowa, USA; 3 The Jackson Laboratory, Bar Harbor, Maine, USA; 4 Schepens Eye Research Institute and Department of Ophthalmology, Harvard Medical School, Boston, Massachusetts, USA; 5 Howard Hughes Medical Institute, Bar Harbor, Maine, USA; 6 Tufts University School of Medicine, Boston, Massachusetts, USA

## Abstract

**Background:**

The *Gpnmb *gene encodes a transmembrane protein whose function(s) remain largely unknown. Here, we assess if a mutant allele of *Gpnmb *confers susceptibility to glaucoma by altering immune functions. DBA/2J mice have a mutant *Gpnmb *gene and they develop a form of glaucoma preceded by a pigment dispersing iris disease and abnormalities of the immunosuppressive ocular microenvironment.

**Results:**

We find that the *Gpnmb *genotype of bone-marrow derived cell lineages significantly influences the iris disease and the elevation of intraocular pressure. GPNMB localizes to multiple cell types, including pigment producing cells, bone marrow derived F4/80 positive antigen-presenting cells (APCs) of the iris and dendritic cells. We show that APCs of DBA/2J mice fail to induce antigen induced immune deviation (a form of tolerance) when treated with TGFβ2. This demonstrates that some of the immune abnormalities previously identified in DBA/2J mice result from intrinsic defects in APCs. However, the tested APC defects are not dependent on a mutant *Gpnmb *gene. Finally, we show that the *Gpnmb *mediated iris disease does not require elevated IL18 or mature B or T lymphocytes.

**Conclusion:**

These results establish a role for *Gpnmb *in bone marrow derived lineages. They suggest that affects of *Gpnmb *on innate immunity influence susceptibility to glaucoma in DBA/2J mice.

## Background

The glaucomas are a common group of potentially blinding diseases that by 2010 will affect approximately 60 million people worldwide [1]. The glaucomas share a clinical phenotype including a progressive degeneration of the optic nerve [2,3]. This glaucomatous optic neuropathy causes a progressive and irreversible loss of vision, and may lead to complete blindness. Significant known risk factors for glaucoma include elevated intraocular pressure (IOP), aging, positive family history, race, abnormal optic nerve head morphology and decreased central corneal thickness [4-12]. Of these, the only currently modifiable risk factor is IOP, which is the target of all existing glaucoma treatments [13-15]. One means of gaining a better understanding of glaucoma pathogenesis, and ultimately the creation of new therapeutic interventions, is to study the underlying molecular pathways with experimental systems such as the mouse. With the recent descriptions of mouse strains and techniques relevant to studying glaucoma, genetic approaches in mice offer great promise for testing new and potentially novel hypotheses related to glaucoma [16-19].

Experiments with DBA/2J (D2) mice have suggested that immune abnormalities may contribute to some forms of glaucoma [20,21]. D2 mice develop a form of glaucoma involving a pigment dispersing iris disease that aberrantly deposits pigment throughout the anterior chamber, including the drainage structures of the eye [22-24]. As a consequence, D2 and several closely related strains develop elevated IOP and glaucomatous neuropathy [24-30]. Eyes of D2 mice also exhibit multiple abnormalities in ocular immune privilege [20], including deficient anterior chamber associated immune deviation (ACAID). Importantly, the iris pigment dispersion component of the D2 iris disease and the inability to support ACAID are simultaneously rescued when their marrow is repopulated with cells from B6D2F1 mice [20]. While the bone marrow origin of immune cells involved in ACAID explains the restoration of ACAID by B6D2F1 bone marrow cells (BMC), the simultaneous resolution of the pigment dispersing iris disease links this disease to bone marrow derived cells. The B6D2F1 mice that served as a source of normal BMC are offspring of a cross between glaucoma prone D2 and normal C57BL/6J (B6) mice. These B6D2F1 mice are heterozygous for all B6 and D2 specific alleles across the entirety of the autosomal genome. Therefore, no specific genes were mechanistically implicated in the recovery of ACAID or the iris disease. The goal of the current experiments is to identify the gene(s) responsible for mediating this BMC contribution to D2 phenotype.

Genetic experiments have previously shown that mutations in two genes digenically promote glaucoma in D2 mice, *Tyrp1 *and *Gpnmb *[22,23]. The *Tyrp1 *gene encodes a relatively well characterized melanosomal enzyme that is required for eumelanogenesis and is localized at the melanosomal membrane. So far there are no reports of either expression or influence of this gene on cell types derived from the bone marrow. Therefore, *Tyrp1 *is unlikely to be relevant in the BMC mediated glaucoma phenotype of D2 mice. Less is known concerning *Gpnmb*. The *Gpnmb *gene is predicted to encode a transmembrane protein with homology to the melanosomal protein, silver (pMel17), but the function(s) of GPNMB remain largely unknown. Interestingly, *Gpnmb *is expressed by some BM derived cell types [31]. Recent studies demonstrate that GPNMB is expressed by macrophages and functions as a negative regulator of macrophage mediated inflammatory responses [32]. In another study, it was reported that GPNMB binds activated T cells. This binding causes inhibition of TCR-induced T-cell activation for both primary and secondary immune responses [33]. Thus, *Gpnmb *is an attractive candidate that potentially influences the BMC mediated phenotypes in D2 mice.

Here, we test the hypothesis that the *Gpnmb *gene contributes to the bone marrow dependent events in D2 glaucoma. Using D2 mice that only differ in *Gpnmb *genotype [34], we demonstrate that BMC lineages containing wild-type *Gpnmb *can rescue both the pigment dispersing iris disease and IOP elevation of D2 mice. Because GPNMB protein localizes to BM derived antigen-presenting cells (APCs) of the iris and to cultured dendritic cells, we assessed potential phenotypes that may be modulated by these APCs. First, we find that APCs of D2 mice are abnormal in that they fail to induce ACAID, this effect is not dependent on *Gpnmb*. Second, we examined aqueous humor levels of IL18. Bone marrow derived APCs in the iris are a potential source of IL18 [35] and IL18 is reported to become elevated in the aqueous humor of D2 mice as disease progresses [21]. In contrast to the previous report [21], we find no evidence for involvement of IL18 in the D2 disease. Surprisingly, we finally show that effectors of adaptive immune responses, mature B or T lymphocytes, are not necessary to mediate the glaucoma inducing iris disease of D2 mice.

## Results

### *Gpnmb *is a candidate for mediating BM derived contributions to D2 ocular disease

**Figure 1:**
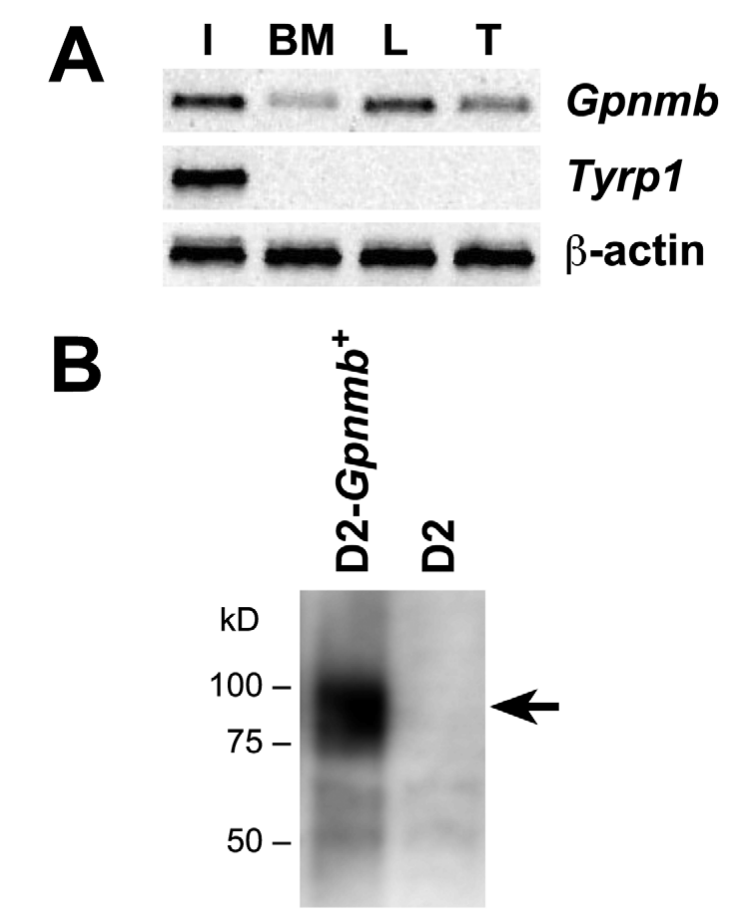
***Gpnmb *is a candidate for mediating BM derived contributions to D2 glaucoma**. **A**). As expected for a candidate capable of mediating the BM derived contributions to the D2 form of glaucoma, *Gpnmb *is expressed in BM derived cells. Agarose gel of designated RT-PCR products amplified from iris (I), bone marrow (BM), lymph node (L), or thymus (T) of normal C57BL/6J mice. (**B**) Iris lysates from D2 and D2-*Gpnmb*^+ ^mice were analyzed on Western blot with anti-GPNMB antibody (B). There is no protein of the expected size for GPNMB (arrow) in the D2 lysate. The faint bands of lower molecular weight likely represent background cross-reactivity of the antibody.

We have previously shown that the D2 iris disease is strongly influenced by a BMC lineage [20]. Our consideration of candidates capable of mediating this influence was based in part on previous genetic experiments demonstrating that the D2 iris disease is initiated by the digenic interaction of the *Tyrp1 *and *Gpnmb *genes [22,23]. To ascertain whether either *Tyrp1 *or *Gpnmb *are plausible candidates for mediating the BMC contributions to D2 iris disease, semi-quantitative RT-PCR was performed to compare gene expression patterns of these genes in iris and bone marrow. Since valid candidates would likely be expressed in cells of the immune system, their expression levels in lymph nodes and thymus were also tested. Supporting a potential role for *Gpnmb *in bone marrow derived phenotypes, expression of *Gpnmb *was detected in all of these tissues. In contrast, the expression of *Tyrp1 *was limited to only the iris (Fig. 1).

Having found that *Gpnmb *was expressed in the appropriate tissues, we next assessed the nature of the *Gpnmb*^*R*150*X *^mutation. The *Gpnmb*^*R*150*X *^mutation in D2 mice creates a premature stop codon. As a result of nonsense-mediated mRNA decay, the mutation is predicted to result in a severe decrease in *Gpnmb *transcript levels [36]. To test this, a quantitative real-time PCR assay for *Gpnmb *was performed. Expression in irides of young predisease D2 mice homozygous for the *Gpnmb*^*R*150*X *^mutation was compared to age and sex matched wild-type controls. With samples normalized to levels of *Rn18s*, the *Gpnmb*^*R*150*X *^mutation resulted in a severe reduction in *Gpnmb *transcript levels (~18 fold, data not shown). In agreement with this, no protein with the expected molecular size was detected by Western analysis using a GPNMB antibody (Fig. 1B). Similar analysis of *Tyrp1 *levels demonstrated no significant change in transcript levels. The expression of *Gpnmb *in immune tissues and the absence of GPNMB protein in R150X mutants support the hypothesis that this mutation disrupts BMC lineage dependent functions of GPNMB and that it may be responsible for bone marrow mediated aspects of the D2 disease.

### A functional requirement for *Gpnmb *in bone marrow

**Figure 2:**
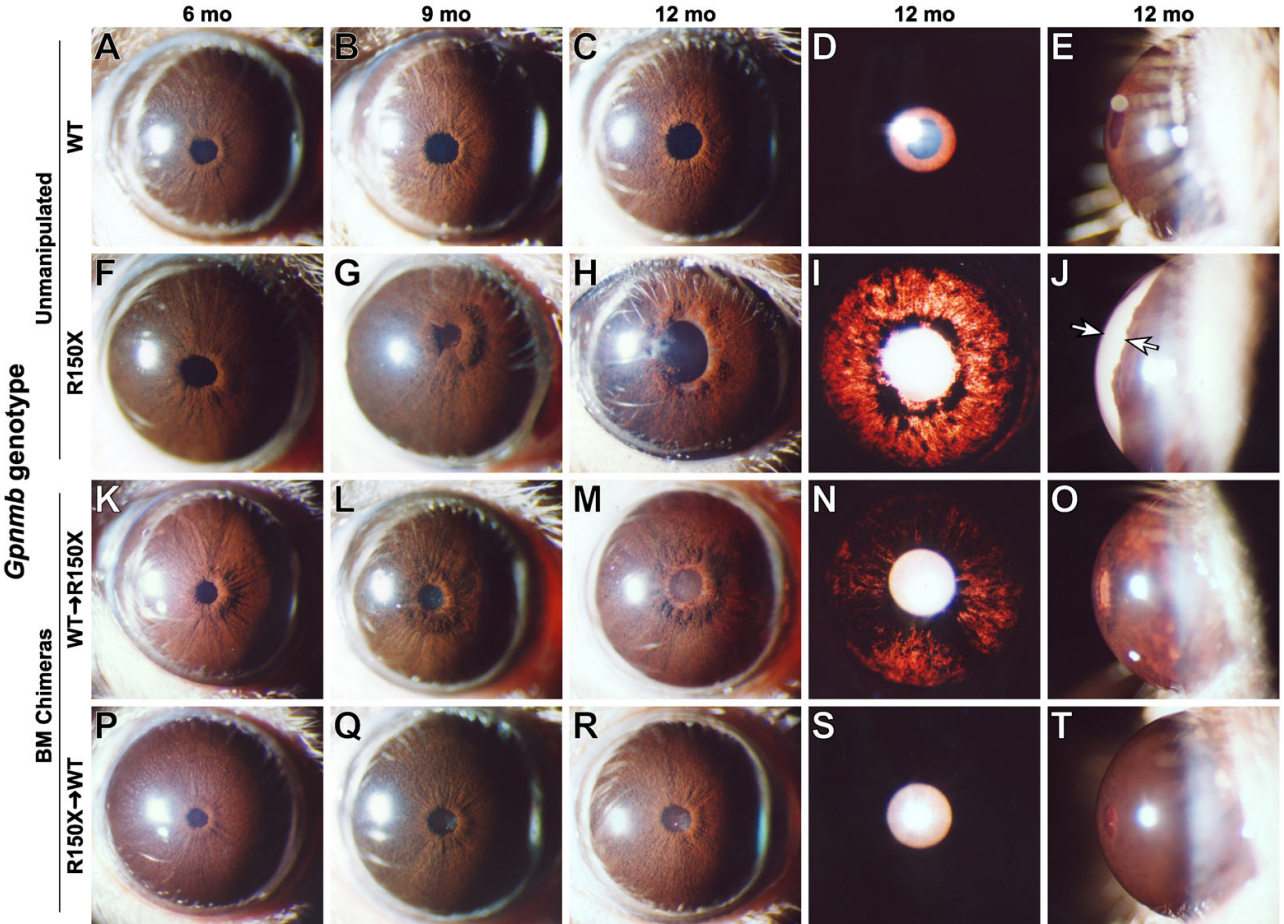
**D2-*Gpnmb*^+ ^bone marrow suppresses iris depigmentation and anterior chamber enlargement**. Representative eyes of the indicated strains and ages are shown. The three left most columns show broad beam illumination to assay for the presence of dispersed pigment within the anterior chamber and iris stromal morphology. The fourth column shows a transilluminating view assaying the degree of iris depigmentation, detectable as red areas within the image where reflected light is passing through the iris. The fifth column show anterior chamber dimensions. Each row represents a different genetic context of either unmanipulated mice (rows 1 & 2) or bone marrow chimeras (rows 3 & 4). (**A to E**) Unmanipulated D2-*Gpnmb*^+ ^mice (WT) exhibit a characteristic iris stromal atrophy phenotype caused by the *Tyrp1*^*b *^mutation that is largely devoid of significant pigment dispersion at all examined ages (1–15 mo). Eyes of D2-*Gpnmb*^+ ^mice do not develop significant transillumination and maintain a normal anterior chamber depth with very little space between the iris and cornea. (**F to J**) Unmanipulated D2 mice (homozygous for the *Gpnmb*^*R*150*X *^mutation) develop a severe pigment dispersing iris disease. At 5–6 mos, the initial stages of iris disease are apparent by a slight peripupillary thickening and subtle changes to the morphology of the iris stroma (note slightly roughened appearance). Subsequently, dispersed pigment becomes aberrantly deposited on a variety of structures including the surface of the iris, lens, and cornea. Dispersed pigment is also deposited in the aqueous humor drainage structures, leading to increased IOP and enlargement of the anterior chamber (space indicated by arrows in J). (**K to O**) D2-*Gpnmb*^+^marrow transferred to D2.*Gpnmb*^*R*150*X *^recipients (WT → R150X) exhibit a pronounced suppression of the typical D2 disease progression. Most notably, the extent of iris degeneration is lessened (compare the peripupillary region of M vs H, and degree of transillumination defects in N vs I) and the anterior chamber does not become enlarged (compare O vs J). (**P to T**) D2 marrow transferred to D2-*Gpnmb*^+ ^recipients (R150X → WT) exhibit no alterations in ocular phenotype compared to unmanipulated D2-*Gpnmb*^+ ^mice.

To genetically test whether the GPNMB genotype of BM derived lineages influences the D2 iris disease, we took advantage of the DBA/2J substrain, D2-*Gpnmb*^+ ^that has a wild-type *Gpnmb *allele but no other known differences to modern D2 mice [34]. Whereas D2 mice with the *Gpnmb*^*R*150*X *^mutation develop an iris disease characterized by significant pigment dispersion, iris atrophy, and anterior chamber enlargement, irides of D2-*Gpnmb*^+ ^mice exhibit far milder phenotypes (Fig. 2A–J). Using these mice as donors and recipients in bone marrow transfers, we next asked whether the genotype of *Gpnmb *in BMC lineages was capable of influencing iris phenotypes.

Lethally irradiated D2 mice were reconstituted with D2-*Gpnmb*^+^bone marrow and followed clinically for indications of iris disease (Fig. 2K–O). In all figures and text, the genotype of donor bone marrow is written first followed by an arrow and then the recipient genotype. For example, WT → R150X where D2-*Gpnmb*^+ ^(WT) bone marrow was transferred into D2 mice homozygous for the R150X mutation. At ages when the D2 iris disease is normally severe, iris phenotypes in D2 mice reconstituted with D2-*Gpnmb*^+ ^bone marrow were significantly rescued toward the wild-type iris phenotype. They developed less pigment dispersion, less transillumination, and less change in the dimensions of the anterior chamber as compared to both unmanipulated D2 mice (compare Fig 2K–O with 2F–J) and D2 mice that were reconstituted with standard D2 marrow that had the *Gpnmb*^*R*150*X *^mutation [20,37]. The converse experiment reconstituting lethally irradiated D2-*Gpnmb*^+ ^mice with standard D2 bone marrow (R150X → WT) was also performed (Fig. 2P–T). Iris phenotypes of D2-*Gpnmb*^+ ^mice reconstituted with standard D2 bone marrow were unaltered and maintained an iris indistinguishable from unmanipulated D2-*Gpnmb*^+ ^mice (compare Fig. 2P–T with 2A–E). In sum, these results indicate that the *Gpnmb*^*R*150*X*^mutation results in the bone-marrow derived portion of the D2 iris disease. However, the influence of the *Gpnmb*^*R*150*X *^mutation acting via bone marrow derived lineages is not itself sufficient toinduce the iris disease in otherwise healthy mice.

### IOP elevation in D2 mice can be modulated by *Gpnmb *bone marrow genotype

**Figure 3:**
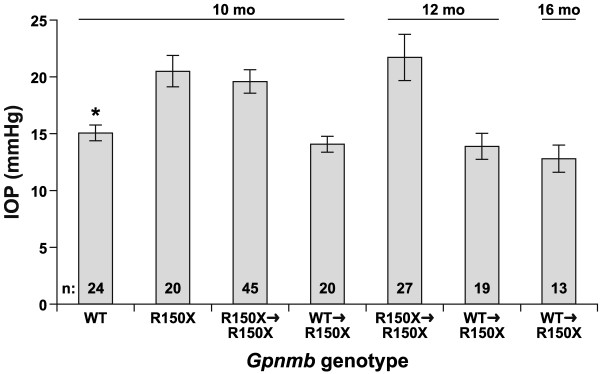
**Bone marrow genotype affects glaucomatous IOP elevation**. Plot of IOP vs *Gpnmb *genotype, with age and numbers of mice indicated. D2-*Gpnmb*^+ ^mice exhibit an average IOP typical of many non-glaucomatous mouse strains. As previously described, D2 mice (homozygous for the *Gpnmb*^*R*150*X *^mutation) exhibit an elevated IOP at 10 mo of age, as do D2 mice receiving syngeneic bone marrow transfers (R150X → R150X). Chimeric mice in which D2-*Gpnmb*^+ ^bone marrow has been transferred into D2 hosts (WT → R150X) continue to maintain non-glaucomatous IOP values, whether examined at 10 mo, or beyond. The IOPs of both *Gpnmb*^+ ^mice and chimeric mice with *Gpnmb*^+ ^bone marrow were significantly lower than those of all mice with *Gpnmb*^*R*150*X *^mutant marrow (P < 0.002 for all comparisons at various ages, *t *test).

In unmanipulated D2 mice, the depigmenting iris disease is typically followed by IOP elevation and enlargement of the anterior chamber. Because the iris disease of D2 mice reconstituted with D2-*Gpnmb*^+ ^bone marrow is ameliorated, and the anterior chamber does not become enlarged, it is likely that these mice do not develop IOP elevation. To directly test this, IOP was measured at multiple ages for bone marrow chimera cohorts and unmanipulated D2 mice of differing *Gpnmb *genotypes (Fig. 3). 10-mo D2-*Gpnmb*^+ ^mice had a relatively normal mean IOP of 16 mmHg, 10-mo unmanipulated D2 mice had an elevated mean IOP of 20 mmHg, and 10-mo D2 mice reconstituted with D2 marrow (R150X → R150X – a syngeneic control for possible influence of bone marrow transfer itself) also had an elevated IOP of 20 mmHg.

Importantly, 10-mo D2 mice reconstituted with D2-*Gpnmb*^+ ^bone marrow (WT → R150X) had a mean IOP of 16 mmHg, indicating that the influence of *Gpnmb *in bone marrow was able to prevent IOP elevation from occurring. Furthermore, the IOP of D2 mice reconstituted with D2-*Gpnmb*^+ ^bone marrow (WT → R150X) remained at these normal levels to at least 16-mo. These results further support the conclusion that the *Gpnmb*^*R*150*X *^mutation influences the glaucoma phenotype of D2 mice via BMC lineages.

### GPNMB localization within F4/80 positive APCs

**Figure 4:**
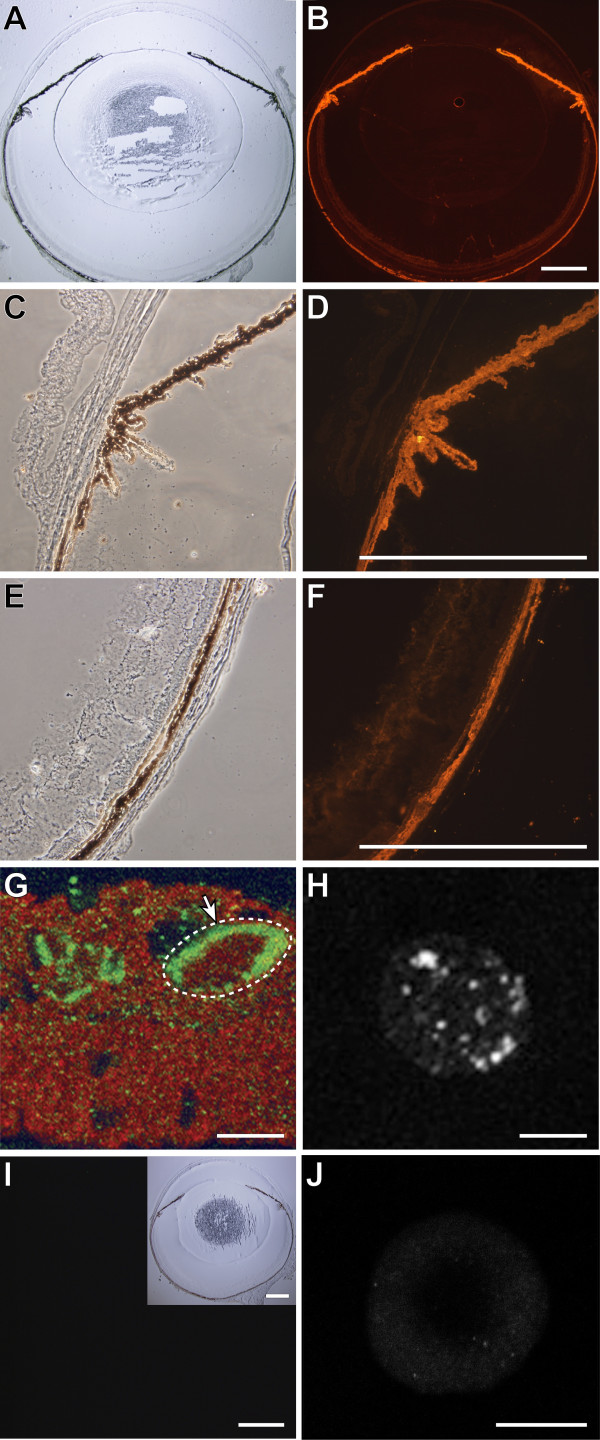
**GPNMB localizes to multiple pigmented tissues of the eye and ocular APCs**. Cryosections of whole eyes from wild-type C57BL/6J mice were imaged in brightfield or phase illumination. GPNMB protein localization was imaged by fluorescent immunohistochemistry and confocal microscopy (red). GPNMB is present in: **(A, B) **multiple pigmented tissues of the eye, **(C, D) **iris and pigmented epithelia of the ciliary body, and **(E, F) **choroid. **(G) **Iris of normal C57BL/6J mouse showing GPNMB (red) within cells labeled by F4/80 (green). An 8 micron Z-series composite demonstrating F4/80 labeling the border of a single cell (dashed white circle) containing GPNMB. **(H) **BM derived dendritic cell from D2-*Gpnmb*^+ ^marrow differentiated in cell culture showing punctate expression of GPNMB, (**I, J**) Negative controls were labelled and imaged with identical conditions and exhibited negligible signal. **(I) **Eyes from D2 mice (homozygous for *Gpnmb*^*R*150*X*^) have no signal for GPNMB, an inset on right hand corner shows the brightfield image of the same section. **(J) **BM derived dendritic cells from D2 mice have no staining for GPNMB. Scale bars A-F, I = 500 μm, G, H, J = 10 μm.

Relatively little is known concerning the *Gpnmb *gene, or it's orthologs in other species [38-40]. Although the ocular localization of GPNMB protein has not previously been described, experiments have suggested two ocular cell types in which GPNMB would likely be found. First, GPNMB is likely to be present in melanin producing cells [22,41,42]. Second, GPNMB is likely to be present in BM derived APCs, including macrophages and a subset of dendritic cells (DCs) [31,32]. In order to begin identifying specific cell types in which *Gpnmb *may function, an anti-GPNMB antibody was used to localize GPNMB in normal adult C57BL/6J mouse eyes (Fig. 4). In adult C57BL/6J mice, we found that GPNMB is robustly detectable within multiple pigmented cells of the eye, including: melanocytes of the iris stroma and pigmented cells of the iris pigment epithelium, the pigmented epithelia of the ciliary body (Fig. 4 A-D), the choroid and to a lesser extent the retinal pigment epithelium (Fig. 4E, F). Diffuse low level labeling was observed throughout the neural retina. GPNMB was not detected in other ocular tissues such as the cornea or lens. In each of the pigmented tissues, GPNMB localization was punctate, likely as a consequence of localization within melanosomes. Though important for other aspects of *Gpnmb *function [43], there is no evidence that any of the pigment producing cells of the eye are derived from the bone marrow. Thus, we next examined whether GPNMB is present in ocular APCs.

The iris normally contains a robust population of APCs [44] and GPNMB has previously been demonstrated to be present in mouse DCs [31]. To determine if a portion of GPNMB in the iris is within APCs, the APC marker F4/80 was tested for co-localization with GPNMB protein. In adult C57BL/6J mice, GPNMB was indeed present within the cytoplasm of iridial F4/80 positive cells (Fig. 4G). To assess the localization of GPNMB in DC lineages, bone marrow progenitors were grown in cell culture, stimulated to differentiate into DCs, and utilized for immunohistochemistry. In DC cells grown in these conditions, GPNMB was observed in intracellular granules (Fig. 4H). Because F4/80 positive APCs and DCs are BM derived cell lineages, this result indicates that the bone marrow functions of *Gpnmb *are likely to be mediated, in part or in whole, by these cells.

### *Gpnmb *deficient APCs fail to induce immune deviation

We next determined the effect of the *Gpnmb *mutation on the ability of APCs to induce immune deviation. The healthy eye exists in a state of immunologic balance that provides immune protection by decreasing risk of immunopathogenic injury to ocular tissues [45]. Anterior chamber associated immune deviation (ACAID) is an anti-inflammatory mechanism that is a form of eye-dependent tolerance mediated by ocular APCs. The immune deviation is initiated by F4/80+ ocular APCs that capture antigen in the eye and migrate via the blood to the spleen [46]. At that site, these APCs generate a population of regulatory T cells capable of inhibiting a Th1-mediated inflammatory immune responses such as a delayed type hypersensitivity response (DTH). We have previously demonstrated that D2 mice lack the ability to support ACAID as evidenced by their inability to inhibit a DTH response, but the genes responsible for this finding are not defined [20].

**Figure 5:**
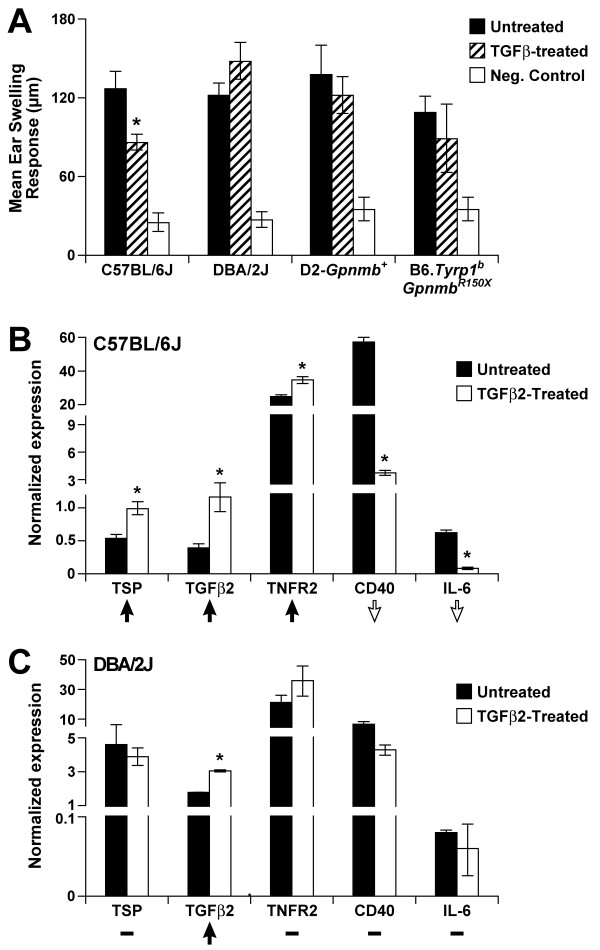
**Tolerogenic capacities and expression profiles of *Gpnmb *deficient APCs**. **(A) **OVA-pulsed APCs isolated from mice of the indicated genotypes were injected into B6D2F1/J recipients. Their ability to induce immune deviation was then measured. Absence of ear swelling indicated functional immune deviation through the suppression of a DTH response to the OVA challenge. We then tested their ability to induce immune deviation as detected by inhibition of DTH i.e. suppression of ear swelling in response to OVA challenge. The experimental summary for each group follows: Untreated (APCs cultured in media, injected into mice, immunization, challenge), TGFβ2-treated (APCs cultured in presence of TGFβ2, injected into mice, immunization, challenge), Neg. Control (challenge only). Induction of immune deviation is detected as the suppression of ear swelling response in the TGFβ2-treated groups as compared to the untreated group. Ear swelling responses are presented as mean ± SEM. (**B-C**) Gene expression profiles of APCs from indicated genotypes. Real-time PCR was employed to determine the expression levels of the indicated genes and expression of each gene was normalized to *Gapdh*. To faciliate visualization of the overall pattern of gene expression changes across these genes, the expression changes in response to TGFβ2-treatment are summarized with an arrow (indicating up or down regulation) or a dash (indicating no change). TSP, thrombospondin 1, official gene name *Thbs1*; TGFβ2, transforming growth factor, beta 2; TNFR2, tumor necrosis factor receptor superfamily, member 1b, official gene name *Tnfrsf1b*; CD40, CD40 antigen; IL-6, interleukin 6.

Non-ocular APCs, such as F4/80+ macrophages derived from thioglycollate-elicited peritoneal exudate cells (PECs), can be converted into APCs that induce immune deviation similar to that induced by ocular APCs by exposing them to TGFβ2 in culture [47-50]. Thus, TGFβ-exposed peripheral APCs resemble ocular APCs in their functional phenotype. To test the *Gpnmb *dependency of this APC phenotype, we compared the ability of APCs with differing *Gpnmb *genotypes to induce immune deviation as detected by inhibition of DTH. To allow experiments with APCs in genetically identical hosts, all experiments used B6D2F1 mice as recipients. TGFβ2-treated APCs from control B6 mice with a wild-type *Gpnmb *allele successfully induced immune deviation that led to inhibition of DTH (Fig. 5A). In contrast, APCs from both *Gpnmb *deficient and sufficient D2 mice failed to induce immune deviation when treated with TGFβ2 (Fig. 5A). Thus, the inability of D2 APCs to mediate immune deviation is not dependent on *Gpnmb *alone. This suggests that some property of D2 APCS other than the *Gpnmb*^*R*150*X *^mutation is responsible for their inability to induce immune deviation.

In parallel with these experiments, we tested the ability of TGFβ-treated APCs from B6.*Tyrp1*^*b*^*Gpnmb*^*R*150*X *^congenic mice to induce tolerance. These congenic mice have the D2-derived *Tyrp1*^*b*^*Gpnmb*^*R*150*X *^mutations on a B6 genetic background (Methods). Also, we evaluated the expression of a panel of genes for which coordinate changes in expression normally contribute to the immune deviation inducing phenotype of APCs [51-53]. Similar to D2 APCs, the B6.*Tyrp1*^*b*^*Gpnmb*^*R*150*X *^derived APCs failed to induce immune deviation when treated with TGFβ2 (Fig. 5A). Arguing against a causative role for the *Tyrp1 *mutation, BALB/c mice have the same *Tyrp1*^*b *^mutation but do not have deficiencies in ACAID [20]. Together, our results suggest that a gene(s) that resides in one of the D2-derived chromosomal regions may be important in mediating APC induced tolerance. (D2-derived chromosomal regions surround the *Tyrp1 *and *Gpnmb *genes in the B6 congenic mice [43]). In agreement with this, the coordinated pattern of changes in gene expression that support immune deviating properties of APCs exhibited a classic tolerizing pattern in B6 mice, but not in D2 (Figure 5B), D2-*Gpnmb*^+ ^or B6.*Tyrp1*^*b*^*Gpnmb*^*R*150*X *^APCs (data not shown).

### Neither *Gpnmb *genotype, nor age, influence aqueous humor levels of IL18

**Figure 6:**
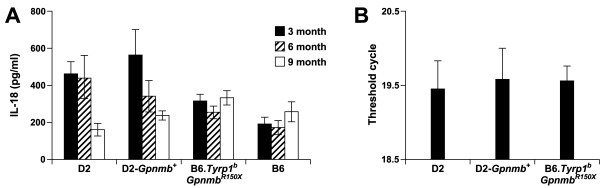
**IL18 levels do not become elevated in D2 aqueous humor or ciliary body**. **(A) **IL18 levels in aqueous humor of age matched D2, D2-*Gpnmb*^+^, B6.*Tyrp1*^*b*^*Gpnmb*^*R*150*X*^, and C57BL/6J mice were determined by ELISA. IL18 levels are expressed as mean +/- SEM from 5 aqueous humor samples in each group. **(B) **Quantitative RT-PCR analysis comparing expression levels of IL18 transcript in ciliary body enriched dissections of 10.5 mo old D2, D2.*Gpnmb*^+ ^and B6.*Tyrp1*^*b*^*Gpnmb*^*R*150*X *^mice. The threshold cycle was calculated with normalization to *Rn18s *and values are expressed as mean +/- SEM, with 3–5 samples in each group.

As an additional step toward defining the potential BM derived functions of *Gpnmb*, we next tested whether *Gpnmb *genotype influences IL18. Secretion of IL18 by macrophages has been implicated in both Th1 and Th2 immune responses. Aqueous humor levels of the immune modulating cytokine IL18 are reported to become elevated in D2 mice, and the levels of IL18 were suggested to associate with increases in IOP [21]. To test whether *Gpnmb *genotype influences IL18 levels, aqueous humor was collected from cohorts of either D2 or B6.*Tyrp1*^*b*^*Gpnmb*^*R*150*X *^mice homozygous for the *Gpnmb*^*R*150*X *^mutation and compared to the strain matched controls, D2-*Gpnmb*^+ ^or C57BL/6J (Fig. 6A). Additionally, expression of IL18 mRNA in tissue enriched in ciliary body was analyzed by real-time PCR (Fig. 6B). In these mice, there was no correlation between *Gpnmb *genotype and IL18 levels. Furthermore, in contrast to previous studies of IL18 in D2 mice [21], we also found no correlation between IL18 and age. Indeed, in our studies, IL18 levels were significantly lower in 9 mo old D2 mice compared to 3 and 6 mo old D2 mice (P < 0.05). These results fail to support a role for IL18 in the D2 form of glaucoma and suggest that the relevant bone marrow derived functions of *Gpnmb *do not influence this aspect of ocular immunity.

### Deficiency of *Rag1 *does not affect *Gpnmb *mediated disease

**Figure 7:**
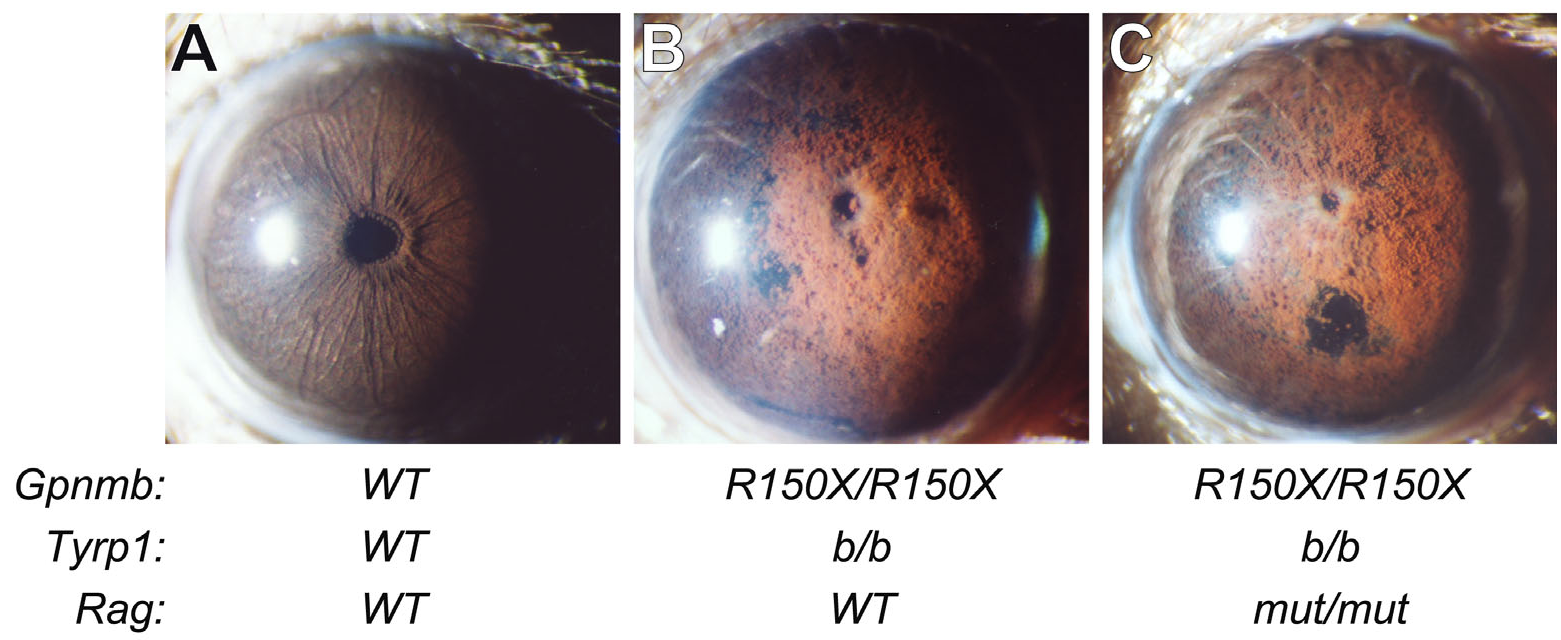
**Loss of adaptive immune reactions has no influence on *Gpnmb *mediated iris disease**. The *Rag1*^*tm*1*Mom *^mutation acts recessively to result in a loss of adaptive immune responses due to lack of mature B and T lymphocytes. Stocks carrying this mutation were bred to B6.*Tyrp1*^*b*^*Gpnmb*^*R*150*X *^mice. Triple mutant mice were isolated and aged, with typical eyes of indicated genotypes shown. The analysis strikingly demonstrates that the immune deficiency associated with *Rag1 *mutation has no effect on *Gpnmb *mediated iris disease. **(A) **Eyes of young B6 mice with wild-type *Tyrp1*, *Gpnmb*, and *Rag1 *alleles have healthy irides. **(B) **At 15 mo, mice mutant for *Tyrp1 *and *Gpnmb *exhibit iris disease characterized by significant pigment dispersion and iris atrophy. n = 10 eyes, age range 14–16 mo.**(C) **At 15 mo, immune deficient mice mutant for *Tyrp1*, *Gpnmb*, and *Rag1 *exhibit an iris disease indistinguishable from immune competent mice on the same genetic background. N = 26 eyes.

GPNMB is present within APCs and APCs initiate and direct adaptive immune responses. Therefore, we tested whether mature B or T lymphocytes, play a role in the *Gpnmb *mediated iris disease. To accomplish this, we exploited a mutation in the recombination activating gene *Rag1*. Mice deficient for *Rag1 *are characterized by arrested development of T and B cells and are therefore incapable of adaptive immune reactions mediated by mature T or B cells. To test the necessity of T and B cells for the pigment dispersing iris disease, we utilized B6.*Tyrp1*^*b*^*Gpnmb*^*R*150*X *^mice [43] with a *Rag1 *mutation. As a consequence of the D2-derived *Tyrp1*^*b *^and *Gpnmb*^*R*150*X *^mutations, B6.*Tyrp1*^*b*^*Gpnmb*^*R*150*X *^mice develop an iris disease with near identical features to that of D2 mice [43]. Ablating the adaptive immune functions of T and B cell had no effect on the iris disease of these mice. The iris disease of *Rag1 *deficient B6.*Tyrp1*^*b*^*Gpnmb*^*R*150*X *^*mice *is indistinguishable to that of their littermates with a functional *Rag1 *gene and an intact adaptive immune system (Fig. 7). Similar results were obtained with mice homozygous for the *Prkdc*^*scid *^mutation that also lack mature B and T lymphocytes [see Additional File 1]. These experiments demonstrate that the loss of adaptive immune reactions has no influence on the age of onset, rate of progression, or final severity of the iris disease mediated by *Tyrp1 *and *Gpnmb *mutations. Given the dependence of disease phenotypes on the *Gpnmb *genotype of bone marrow but not on adaptive immunity, these results suggest that GPNMB may impact innate immune processes. Therefore, alterations of innate immunity may participate in the pathogenesis of the iris disease.

## Discussion

D2 mice develop a pigmentary form of glaucoma involving a pigment dispersing iris disease, increased IOP, and degeneration of retinal ganglion cells [22-24,26]. In recent years, we have begun to study early events within the anterior chamber that initiate the iris disease and IOP elevation [20,22,23,43] as well as later events associated with the neurodegeneration [37,54,55]. We have previously identified two genes responsible for the initiation of this disease, *Tyrp1 *and *Gpnmb*, both of which encode melanosomal proteins [22,42,56]. We have also shown that a non-melanosomal component contributes to the D2 iris disease which acts via a BMC lineage [20]. Here, we have identified the *Gpnmb *gene as an important gene influencing these bone marrow derived contributions.

From the sum of prior and present experiments (see Additional File 2 and refs [20,22]), an attractive hypothesis suggests that the D2 iris disease initially involves a melanosomal defect mediated by the *Tyrp1 *and *Gpnmb *genes that mildly damages the iris and causes cellular debris, including pigment, to be shed into the anterior chamber. Due to deficiency of GPNMB, a bone marrow derived lineage(s) that would normally express *Gpnmb *then reacts abnormally to the iris debris. This results in an inflammatory attack on the iris, severe iris atrophy and the ensuing glaucoma. The abnormally responding bone marrow derived lineage may either fail to inhibit ocular inflammation or may actively promote an inflammatory attack on the iris. This model accounts for the ability of B6D2F1 [20] or D2-*Gpnmb*^+ ^(Fig. 2) bone marrow to suppress the D2 iris disease (because bone derived cells with wild-type *Gpnmb *alleles do not respond abnormally to the debris resulting from the melanosomal insults and mild iris damage). Additionally, this model explains why bone marrow from D2 mice with the *Gpnmb*^*R*150*X *^mutation is not sufficient to confer the severe iris disease when transplanted into recipient mice with wild-type *Gpnmb *alleles (because the recipients with wild-type *Gpnmb *alleles do not have sufficient iris damage to prime the immune system attack of the iris.). Thus, this model, explains why the bone marrow derived function of *Gpnmb *is a necessary, but not sufficient, component of the overall disease.

The functions of *Gpnmb *in BMC derived lineage(s) that are relevant to DBA/2J glaucoma remain unknown. Although *Gpnmb *genotype did not influence the ability of D2 APCs to mediate tolerance to OVA when they were first exposed to TGFβ2 and OVA in culture, GPNMB may still modulate some aspects of APCs that contribute to D2 phenotype and were not assessed. These functions may include their ability to process and present antigens derived from damaged iris cells, their ability to secrete various chemokines that recruit proinflammatory cells or their ability to respond to anti-inflammatory molecules in the ocular microenvironment. Regardless, we show that the immune abnormalities contributing to the *Gpnmb *mediated iris disease do not require functions of the adaptive immune system. This suggests that GPNMB deficiency may influence innate immune cells that are of bone marrow origin and are likely to respond to abnormalities in the iris. Experimental expression of GPNMB in activated macrophage causes reduced release of proinflammatory cytokines such as IL6, IL12 and TNF alpha [32]. We localize GPNMB within APCs. Therefore, it is possible that the *Gpnmb*^*R*150*X *^mutation alters APCs so that they are more prone to mediate inflammation and this abnormal phenotype promotes ocular inflammation in D2 mice.

Other groups have previously localized GPNMB in macrophages and in the MHC class II compartment of DCs [31]. The punctate localization of GPNMB in pigmented cells and its melanosomal targeting sequence are consistent with another function of GPNMB in melanosomes [22]. Interestingly, melanosomes and the MHC class II compartment are both lysosome-related organelles [57,58]. This correlation suggests that similar to molecules such as BLOC complexes and LYST [59,60], GPNMB may be a molecule that functions in multiple classes of lysosome-related organelles.

The glaucomatous potential of the DBA/2J iris disease has recently been shown to be strikingly sensitive to genetic background [43]. When transferred to the C57BL/6J genetic background, the *Gpnmb*^*R*150*X *^and *Tyrp1*^*b *^mutations result in a severe pigment dispersing iris disease that is phenotypically indistinguishable from the iris disease of D2 mice. Surprisingly, however, these B6 mice are resistant to IOP elevation and glaucoma. Thus, there are additional modifier genes whose alleles determine whether or not IOP becomes elevated and glaucoma ensues. Although these modifier genes remain to be identified, our preliminary data suggest existence of at least 3 genetic loci, on different chromosomes to *Gpnmb *and *Tyrp1*, that impact the nature of the anterior chamber disease and determine if IOP becomes elevated. Given the dependence of the iris disease on bone marrow genotype, at least some of these modifiers are likely to influence immune functions. Since the chromosomal region containing either *Gpnmb *or *Tyrp1 *transferred the inability of APCs to mediate tolerance to the B6 congenic strain, there must be another strain specific allele that influences APC functions. Efforts to identify these strain specific modifier alleles are underway and are likely to shed additional light on the molecular basis of immune contributions to this glaucoma. Characterizing these genes is expected to improve understanding of APC responses within the anterior chamber and mechanisms of inducing tolerance.

## Conclusion

We have demonstrated that *Gpnmb *influences the glaucoma phenotype of D2 mice by a BM derived mechanism that does not require adaptive immunity. Although immunity has not yet been shown to contribute to human pigmentary glaucoma, the inflammation is subclinical in the mice and may have been undetectable on standard examination of patient's eyes. Thus, similar mechanisms of mild inflammation may be active in human pigmentary glaucoma and perhaps other ocular diseases in which pigment dispersal occurs within the anterior chamber. These findings represent an important step toward gaining a molecular understanding of the mechanisms active in this pigmentary form of glaucoma

## Methods

### Animal husbandry

D2 mice were obtained from The Jackson Laboratory, Bar Harbor, Maine. Some studies also utilized B6.D2-*Tyrp1*^*b*^*Gpnmb*^*R*150*X*^/Sj mice (referred to as B6 *Tyrp1*^*b*^*Gpnmb*^*R*150*X*^), an N10 congenic strain of mice which are homozygous for chromosomal intervals containing the D2-derived *Tyrp1*^*b *^and *Gpnmb*^*R*150*X *^mutations that have been backcrossed into the C57BL/6J genetic background [43]. Mice with a recombination activating gene 1 mutation (*Rag1*^*tm*1*Mom*^) were obtained from The Jackson Laboratory and crossed to the B6.*Tyrp1*^*b *^*Gpnmb*^*R*150*X *^strain to produce mutants that were homozygous for all three mutations (B6.Cg-*Rag1*^*tm*1*Mom*^*Tyrp1*^*b*^*Gpnmb*^*R*150*X*^/Sj, referred to as *Rag1 *mutant B6 *Tyrp1*^*b*^*Gpnmb*^*R*150*X*^). Two strains of control mice with wild-type *Gpnmb *alleles were utilized in these studies, DBA/2J with a wild-type *Gpnmb *allele (DBA/2J-*Gpnmb*^+^/Sj, referred to as D2-*Gpnmb*^+^) [34] and C57BL/6J. Since the *Gpnmb*^+^allele is the ancestral D2 allele, the *Gpnmb*^*R*150*X *^mutation is the only known genetic difference between the D2 and D2-*Gpnmb*^+ ^strains [22]. C57BL/6J mice (with wild-type *Tyrp1 *and *Gpnmb *alleles) were obtained from The Jackson Laboratory. All D2 background mice were maintained on a 6% fat NIH 31 diet provided *ad libitum*, and the water was acidified to pH 2.8–3.2. All B6 background mice were similarly maintained but on an NIH 31 diet with 4% fat content. Mice were housed in cages containing white pine bedding and covered with polyester filters. The environment was kept at 21°C with a 14-h light:10-h dark cycle. All animals were treated according to the guidelines of the Association for Research in Vision and Ophthalmology for use of animals in research. All experimental protocols were approved by the Animal Care and Use Committee of The Jackson Laboratory or The University of Iowa.

### Generation and analysis of bone marrow chimeras

Bone marrow chimeras were generated as follows: 4–8 wk old female D2 and D2-*Gpnmb*^+ ^mice were lethally irradiated (1000 rad from a ^137^Cs source) and then received 200 μl intravenous injections containing 5 × 10^6 ^T cell depleted bone marrow cells from the indicated donor strains. Donor marrow was depleted of T lymphocytes with 10 μg/mL of purified monoclonal antibodies to CD4 (GK1.5; The Jackson Laboratory Flow Cytometry Service, Bar Harbor, Maine) and CD8a (53–6.72; The Jackson Laboratory Flow Cytometry Service). Eyes of chimeras were clinically assayed at 2–3 mo intervals.

### Intraocular pressure measurement

IOP was measured using the microneedle method as previously described in detail [61,62]. Because the IOPs of B6 mice are very consistent, B6 mice were interspersed with experimental mice during all experiments as a methodological control to ensure proper equipment calibration and performance.

### Slit-lamp examination

Eyes were examined with a slit-lamp bio-microscope and photographed with a 40× objective lens. Phenotypic assessment of iris disease was determined by indices of iris atrophy dispersed pigment and transillumination defects following previously described criteria [20,22-24]. Transillumination is an assay of iris disease whereby reflected light passing through depigmented areas of iris tissue are visualized as red light.

### RNA Isolation and Quantitative RT-PCR

Tissues were dissected, homogenized, and total RNA extracted. cDNA was generated using 300 ng of total RNA isolated from dissected irides or 70 ng of total RNA isolated from ciliary body enriched dissections. Real-time PCR data were collected utilizing standard reaction conditions, with primer efficiencies determined from serial dilutions of cDNA and relative expression calculated as previously published [63-65]. Reaction conditions and primer sequences are available upon request.

### Western blotting

Iris tissue from D2 and D2-*Gpnmb*^+ ^was homogenized in lysis buffer (10 mM Tris pH 7.6, 150 mM NACl, 1% Triton X-100 and supplemented with protease inhibitors). The lysate was resolved on a 4–20% SDS-PAGE gradient gel. Proteins were blotted to a PVDF membrane, followed by incubation with anti-GPNMB antibody (R&D systems), secondary antibody and detected using SuperSignal West Pico chemiluminescent substrate (Pierce Biotechnology).

### Immunohistochemistry

Enucleated eyes were embedded in Optimal Cutting Temperature embedding medium (Tissue-Tek O.C.T. Compound, Sakura Finetek U.S.A., Inc., Torrance, CA), seven-micrometer sections cut, and sections transferred to glass slides (CryoJane, Instrumedics, Inc., St. Louis, MO). Cryosections were air dried for 30 min at room temperature, fixed for 5 min in ice-cold acetone, again air dried for 30 min at room temperature, and rehydrated in PBS for 5 min. Sections were blocked 1 hr at room temperature with 10% normal donkey serum and 30 mg/mL BSA in PBS. Primary antibodies were applied for 1 hr at room temperature using polyclonal rabbit anti-GPNMB antibody (diluted 1:200; R&D Systems Inc., Minneapolis, MN) or anti-F4/80 (diluted 1:200; Serotec Inc., Raleigh, NC). Primary antibody was removed by three washes (5 min each) in PBS and the sections were treated for 1 hr at room temperature with AlexaFluor conjugated secondary antibodies (1:200 dilution, Invitrogen-Molecular Probes, Carlsbad, CA) diluted in 1% normal donkey serum and 10 mg/mL BSA in PBS. After three washes in PBS, the sections were mounted (Vectashield, Vector Laboratories, Burlingame, CA) and viewed by fluorescence microscopy. All photomicrographs were taken with identical camera settings. For studies of bone marrow derived dendritic cell cultures, cells were fixed in 4% paraformaldehyde for 20 min at room temperature. Cells were permeabilized with PBS containing 0.1% Triton X-100, incubated with polyclonal rabbit anti-GPNMB antibody (diluted 1:200; R&D Systems Inc., Minneapolis, MN) for 1 hr at room temperature, washed three times in PBS, and treated for 1 hr at room temperature with FITC conjugated secondary antibodies (1:200 dilution, Jackson Immunoresearch, West Grove, PA). After three washes in PBS, the cells were mounted in fluorescent mounting medium (Vectashield, Vector Laboratories, Burlingame, CA) and viewed by confocal microscopy.

### Bone marrow derived dendritic cell cultures

BM derived cells obtained from D2 or D2-*Gpnmb*^+ ^mouse femur were cultured in the presence of GM-CSF (20 ng/ml) in 24 well plates (Costar Corp., Cambridge, MA). The nonadherent cells were removed from the culture and replated with fresh medium containing GM-CSF every alternate day. On day 10, cells were harvested and a subset analysed by flow cytometry. In the current experiments, >95% of cells labeled positive for the mouse APC marker CD11c.

### Analysis of aqueous humor IL18

Aqueous humor was collected as described previously [21]. IL18 protein level in the aqueous humor was measured using a commercially available ELISA assay following the manufacturer's recommended protocol (Bender MedSystems, San Bruno, CA). Since overall protein levels in D2 aqueous humor are increased in older mice (at 6 and 9 mo) [20], samples were compared by analyzing equal volumes of aqueous humor rather than normalizing to total protein.

### Assay for immune deviation

Peritoneal exudate cells (PECs) were obtained 3 days following 2 ml intra-peritoneal injections of a 3% thioglycollate solution (Sigma-Aldrich, St. Louis, MO). Plastic adherent macrophages from these cells were used as APCs (>95% F4/80+). Cells were cultured overnight (1 × 10^5^/well) in a 96-well plate in the presence of TGFβ2 (R&D Systems, CA, USA, 5 ng/ml final concentration) in serum free culture media, and pulsed with ovalbumin antigen (OVA) (7 mg/ml). Adherent cells were harvested, after washing off culture medium with cold Hank's balanced salt solution, and intravenously injected into B6D2F1 recipients (5 × 10 ^3 ^cells/mouse). Seven days following, recipients were immunized subcutaneously into the nape of the neck with OVA/CFA (50 μg). Animals that did not receive any APCs or immunization served as a negative control. A week later, animals in all groups received intra-dermal inoculation of OVA (200 μg/20 μl) into their right ear pinna. The left ear served as an untreated control. Thickness of both ears was measured immediately before and at 24-h interval after the OVA injection using a micrometer (Mitutoyo 227-101, MTI Corp., Paramus, NJ, USA). The measurements were performed in triplicates. Delayed type hypersensitivity (DTH) was measured as ear swelling [(24-h measurement – 0-h measurement in the experimental ear) – (24-h measurement – 0-h measurement in the untreated control ear)]. Immune deviation was detected as the suppression in DTH in the recipients of TGFβ2 treated APCs as compared to those infused with untreated APCs. A two-tailed Student's t-test was used with significance assumed at P < 0.05. DTH results were confirmed by repeating the experiments a second time.

### Serum free medium

Serum-free medium (SFM) was used for in vitro assays. The medium contained: RPMI-1640, 10 mM HEPES, 0.1 mM NEAA, 1 mM Sodium pyruvate, 100 U/ml Penicillin, 100 mg/ml Streptomycin (Bio Whittaker, Walksville, MD), 0.1% BSA (Sigma-Aldrich, St. Louis, MO) and ITS+ culture supplement [1 μg/ml iron-free transferrin, 10 ng/ml linoleic acid, 0.3 ng/ml Na_2_Se and 0.2 μg/ml Fe(NO_3_)_3_] (Collaborative Biomedical Products, Bedford, MA).

### Genetic nomenclature and stocks

The official full names of genes (with abbreviations in parentheses), alleles, and stocks utilized in this study are as follows. 1. Glycoprotein (transmembrane) nmb (*Gpnmb*). The *ipd *allele of *Gpnmb *results from the R150X premature stop codon mutation, *Gpnmb*^*R*150*X *^[22]. 2. Tyrosinase-related protein 1 (*Tyrp1*). The *b *allele of *Tyrp1 *encodes two amino acid substitutions compared to the C57BL/6J-derived allele [56]. This D2 allele is also referred to as *isa *in the mouse genome database and elsewhere [22,23]. The DBA/2J stock utilized here was stock number 000671 from The Jackson Laboratory. 3. Recombination activating gene 1 (*Rag1*). The *tm1Mom *mutation is a targeted knock-out [66]. The *Rag1*^*tm*1*Mom *^stock utilized here was B6.129S7-*Rag1*^*tm*1*Mom*^/J (stock 002216 from The Jackson Laboratory). 4. Protein kinase, DNA activated, catalytic polypeptide (*Prkdc*). The *scid *mutation arose spontaneously in the CB17 strain and has since been backcrossed onto C57BL/6J genetic background [67]. The *Prkdc*^*scid *^stock utilized here was B6.CB17-*Prkdc*^*scid*^/SzJ (stock 001913 from The Jackson Laboratory).

## Authors' contributions

MGA, KSN, SM and SWMJ conceived experiments, analysed the data and prepared the manuscript. SWMJ oversaw all aspects of the study. MGA bred and performed clinical analysis of mice. KSN performed Western, ELISA and bone marrow derived dendritic cell culture experiments. LAA and CT performed tissue sectioning and immunohistochemistry. AM performed quantitative PCR. SM performed the ACAID and quantitative PCR experiments using PECs.

## Supplementary Material

Additional file 1 **Loss of adaptive immune reactions has no influence on the age of onset, rate of progression, or final severity of the *Tyrp1 *or *Gpnmb *mediated iris disease**. The *Rag1*^*tm*1*Mom *^and *Prkdc*^*scid *^mutations both act recessively and result in essentially identical phenotypes characterized by loss of adaptive immune responses due loss of mature B and T lymphocytes. Stocks carrying each of these mutations were separately bred to B6.*Tyrp1*^*b*^*Gpnmb*^*R*150*X *^mice. During the course of establishing triple mutant stocks, single and double mutant progeny classes were also isolated. Subsets of these were aged, with typical eyes of indicated genotypes shown. The combined analysis strikingly demonstrates that the immune deficiency associated with *Rag1 *or *Prkdc *mutation has no effect on *Tyrp1 *or *Gpnmb *mediated iris disease. Each column corresponds to a particular genotype with respect to *Tyrp1 *and *Gpnmb*; the rows are organized according to *Rag1 *or *Prkdc *genotype and age (homozygous mutants labeled as "Immune Deficient"; heterozygous and wild-type mice labeled "Normal"). (**A to D**) Eyes of young mice of all genotypes are characterized by healthy irides. By 8–9 mo, *Tyrp1 *and *Gpnmb *mutant mice exhibited typical signs of early disease that did not differ whether the mice were (**E to H**) immune competent, or (**I to L**) immune deficient. At 14+ mo, *Tyrp1 *and *Gpnmb *mutant mice exhibited signs of late disease that did not differ whether the mice were (**M to P**) immune competent or (**Q to T**) immune deficient. Panels K and Q are based on observations of 2 eyes, panel I from 6 eyes, and all other panels from groups of 8 to 26 eyes. Click here for file

Additional file 2 ***Gpnmb*^*R*150*X *^mutation does not directly elevate IOP**. Mean IOP ± SEM is presented. For ease of comparison, some of the data are duplicated from Figure 3. It is clear that the *Gpnmb*^*R*150*X *^mutation contributes to the iris disease of DBA/2J mice and is a critical component of the glaucoma. Manipulations that prevent severe iris disease in DBA/2J mice prevent both anterior chamber enlargement (a marker of IOP elevation) and glaucoma development [20,22]. Thus, the iris disease is an essential component of the glaucoma. To determine if the *Gpnmb*^*R*150*X *^mutation also influences IOP independently of the iris disease, we have assessed IOP in aged DBA/2J mice that have the *Gpnmb *mutation in all tissues but that do not develop the severe iris disease. These mice were homozygous for the wild-type C57BL/6J allele of *Tyrp1 *(*Tyrp1*^*B*6^, that was backcrossed into DBA/2J for more than 10 generations) [34] and the mutant DBA/2J allele of *Gpnmb *(*Gpnmb*^*D*2 ^= *Gpnmb*^*R*150*X*^). Since mutant DBA/2J alleles of both *Tyrp1 *and *Gpnmb *are necessary to develop severe iris disease, the *Tyrp1*^*B*6^*Gpnmb*^*D*2 ^mice did not do so. Importantly and despite the presence of the *Gpnmb *mutation in the iris and bone marrow, the *Tyrp1*^*B*6 ^*Gpnmb*^*D*2 ^mice did not develop elevated IOP. Thus, the *Gpnmb*^*R*150*X *^mutation does not induce high IOP directly but does so by inducing the iris disease. Click here for file

## References

[B1] Quigley HA, Broman AT (2006). The number of people with glaucoma worldwide in 2010 and 2020. Br J Ophthalmol.

[B2] Allingham RR, Damji KF, Freedman S, Moroi SE, Shafranov G, Shields MB (2004). Shields' Textbook of Glaucoma.

[B3] Nickells RW, Jampel HD, Zack DJ, Rimoin DL, Conner MJ, Pyeritz RE, Korf BR (2002). Glaucoma. Emery & Rimoins Principles and Practices of Medical Genetics.

[B4] Gordon MO, Beiser JA, Brandt JD, Heuer DK, Higginbotham EJ, Johnson CA, Keltner JL, Miller JP, Parrish RK, Wilson MR, Kass MA (2002). The Ocular Hypertension Treatment Study: baseline factors that predict the onset of primary open-angle glaucoma. Arch Ophthalmol.

[B5] Kass MA, Heuer DK, Higginbotham EJ, Johnson CA, Keltner JL, Miller JP, Parrish RK, Wilson MR, Gordon MO (2002). The Ocular Hypertension Treatment Study: a randomized trial determines that topical ocular hypotensive medication delays or prevents the onset of primary open-angle glaucoma. Arch Ophthalmol.

[B6] Mitchell P, Smith W, Attebo K, Healey PR (1996). Prevalence of open-angle glaucoma in Australia. The Blue Mountains Eye Study. Ophthalmology.

[B7] Quigley HA (2005). Glaucoma: macrocosm to microcosm the Friedenwald lecture. Invest Ophthalmol Vis Sci.

[B8] Sommer A, Tielsch JM, Katz J, Quigley HA, Gottsch JD, Javitt J, Singh K (1991). Relationship between intraocular pressure and primary open angle glaucoma among white and black Americans. The Baltimore Eye Survey. Arch Ophthalmol.

[B9] Tielsch JM, Katz J, Singh K, Quigley HA, Gottsch JD, Javitt J, Sommer A (1991). A population-based evaluation of glaucoma screening: the Baltimore Eye Survey. Am J Epidemiol.

[B10] Tielsch JM, Katz J, Sommer A, Quigley HA, Javitt JC (1994). Family history and risk of primary open angle glaucoma. The Baltimore Eye Survey. Arch Ophthalmol.

[B11] Weinreb RN, Khaw PT (2004). Primary open-angle glaucoma. Lancet.

[B12] Wolfs RC, Klaver CC, Ramrattan RS, van Duijn CM, Hofman A, de Jong PT (1998). Genetic risk of primary open-angle glaucoma. Population-based familial aggregation study. Arch Ophthalmol.

[B13] Clark AF, Yorio T (2003). Ophthalmic drug discovery. Nat Rev Drug Discov.

[B14] Quigley HA (2005). New paradigms in the mechanisms and management of glaucoma. Eye.

[B15] Schwartz K, Budenz D (2004). Current management of glaucoma. Curr Opin Ophthalmol.

[B16] Goldblum D, Mittag T (2002). Prospects for relevant glaucoma models with retinal ganglion cell damage in the rodent eye. Vision Res.

[B17] John SWM (2005). Mechanistic insights into glaucoma provided by experimental genetics the cogan lecture. Invest Ophthalmol Vis Sci.

[B18] John SWM, Anderson MG, Smith RS (1999). Mouse genetics: a tool to help unlock the mechanisms of glaucoma. J Glaucoma.

[B19] Weinreb RN, Lindsey JD (2005). The importance of models in glaucoma research. J Glaucoma.

[B20] Mo JS, Anderson MG, Gregory M, Smith RS, Savinova OV, Serreze DV, Ksander BR, Streilein JW, John SWM (2003). By altering ocular immune privilege, bone marrow-derived cells pathogenically contribute to DBA/2J pigmentary glaucoma. J Exp Med.

[B21] Zhou X, Li F, Kong L, Tomita H, Li C, Cao W (2005). Involvement of inflammation, degradation, and apoptosis in a mouse model of glaucoma. J Biol Chem.

[B22] Anderson MG, Smith RS, Hawes NL, Zabaleta A, Chang B, Wiggs JL, John SWM (2002). Mutations in genes encoding melanosomal proteins cause pigmentary glaucoma in DBA/2J mice. Nat Genet.

[B23] Chang B, Smith RS, Hawes NL, Anderson MG, Zabaleta A, Savinova O, Roderick TH, Heckenlively JR, Davisson MT, John SWM (1999). Interacting loci cause severe iris atrophy and glaucoma in DBA/2J mice. Nat Genet.

[B24] John SWM, Smith RS, Savinova OV, Hawes NL, Chang B, Turnbull D, Davisson M, Roderick TH, Heckenlively JR (1998). Essential iris atrophy, pigment dispersion, and glaucoma in DBA/2J mice. Invest Ophthalmol Vis Sci.

[B25] Bayer AU, Neuhardt T, May AC, Martus P, Maag KP, Brodie S, Lutjen-Drecoll E, Podos SM, Mittag T (2001). Retinal morphology and ERG response in the DBA/2NNia mouse model of angle-closure glaucoma. Invest Ophthalmol Vis Sci.

[B26] Libby RT, Anderson MG, Pang IH, Robinson ZH, Savinova OV, Cosma IM, Snow A, Wilson LA, Smith RS, Clark AF, John SWM (2005). Inherited glaucoma in DBA/2J mice: pertinent disease features for studying the neurodegeneration. Vis Neurosci.

[B27] Sheldon WG, Warbritton AR, Bucci TJ, Turturro A (1995). Glaucoma in food-restricted and ad libitum-fed DBA/2NNia mice. Lab Anim Sci.

[B28] Danias J, Lee KC, Zamora MF, Chen B, Shen F, Filippopoulos T, Su Y, Goldblum D, Podos SM, Mittag T (2003). Quantitative analysis of retinal ganglion cell (RGC) loss in aging DBA/2NNia glaucomatous mice: comparison with RGC loss in aging C57/BL6 mice. Invest Ophthalmol Vis Sci.

[B29] Inman DM, Sappington RM, Horner PJ, Calkins DJ (2006). Quantitative correlation of optic nerve pathology with ocular pressure and corneal thickness in the DBA/2 mouse model of glaucoma. Invest Ophthalmol Vis Sci.

[B30] Schlamp CL, Li Y, Dietz JA, Janssen KT, Nickells RW (2006). Progressive ganglion cell loss and optic nerve degeneration in DBA/2J mice is variable and asymmetric. BMC Neurosci.

[B31] Shikano S, Bonkobara M, Zukas PK, Ariizumi K (2001). Molecular cloning of a dendritic cell-associated transmembrane protein, DC-HIL, that promotes RGD-dependent adhesion of endothelial cells through recognition of heparan sulfate proteoglycans. J Biol Chem.

[B32] Ripoll VM, Irvine KM, Ravasi T, Sweet MJ, Hume DA (2007). Gpnmb is induced in macrophages by IFN-gamma and lipopolysaccharide and acts as a feedback regulator of proinflammatory responses. J Immunol.

[B33] Chung JS, Sato K, Dougherty, Cruz PD, Ariizumi K (2007). DC-HIL is a negative regulator of T lymphocyte activation. Blood.

[B34] Howell GR, Libby RT, Marchant JK, Wilson LA, Cosma IM, Smith RS, Anderson MG, John SWM (2007). Absence of glaucoma in DBA/2J mice homozygous for wild-type versions of Gpnmb and Tyrp1. BMC Genet.

[B35] Boraschi D, Dinarello CA (2006). IL-18 in autoimmunity: review. Eur Cytokine Netw.

[B36] Maquat LE (2005). Nonsense-mediated mRNA decay in mammals. J Cell Sci.

[B37] Anderson MG, Libby RT, Gould DB, Smith RS, John SW (2005). High-dose radiation with bone marrow transfer prevents neurodegeneration in an inherited glaucoma. Proc Natl Acad Sci USA.

[B38] Owen TA, Smock SL, Prakash S, Pinder L, Brees D, Krull D, Castleberry TA, Clancy YC, Marks SC, Safadi FF, Popoff SN (2003). Identification and characterization of the genes encoding human and mouse osteoactivin. Crit Rev Eukaryot Gene Expr.

[B39] Safadi FF, Xu J, Smock SL, Rico MC, Owen TA, Popoff SN (2001). Cloning and characterization of osteoactivin, a novel cDNA expressed in osteoblasts. J Cell Biochem.

[B40] Turque N, Denhez F, Martin P, Planque N, Bailly M, Begue A, Stehelin D, Saule S (1996). Characterization of a new melanocyte-specific gene (QNR-71) expressed in v-myc-transformed quail neuroretina. Embo J.

[B41] Bachner D, Schroder D, Gross G (2002). mRNA expression of the murine glycoprotein (transmembrane) nmb (Gpnmb) gene is linked to the developing retinal pigment epithelium and iris. Brain Res Gene Expr Patterns.

[B42] Le Borgne R, Planque N, Martin P, Dewitte F, Saule S, Hoflack B (2001). The AP-3-dependent targeting of the melanosomal glycoprotein QNR-71 requires a di-leucine-based sorting signal. J Cell Sci.

[B43] Anderson MG, Libby RT, Mao M, Cosma IM, Wilson LA, Smith RS, John SWM (2006). Genetic context determines susceptibility to intraocular pressure elevation in a mouse pigmentary glaucoma. BMC Biol.

[B44] McMenamin PG, Crewe J, Morrison S, Holt PG (1994). Immunomorphologic studies of macrophages and MHC class II-positive dendritic cells in the iris and ciliary body of the rat, mouse, and human eye. Invest Ophthalmol Vis Sci.

[B45] Streilein JW (2003). Ocular immune privilege: the eye takes a dim but practical view of immunity and inflammation. J Leukoc Biol.

[B46] Williamson JS, Bradley D, Streilein JW (1989). Immunoregulatory properties of bone marrow-derived cells in the iris and ciliary body. Immunology.

[B47] Hara Y, Caspi RR, Wiggert B, Dorf M, Streilein JW (1992). Analysis of an in vitro-generated signal that induces systemic immune deviation similar to that elicited by antigen injected into the anterior chamber of the eye. J Immunol.

[B48] Hara Y, Okamoto S, Rouse B, Streilein JW (1993). Evidence that peritoneal exudate cells cultured with eye-derived fluids are the proximate antigen-presenting cells in immune deviation of the ocular type. J Immunol.

[B49] Wilbanks GA, Mammolenti M, Streilein JW (1992). Studies on the induction of anterior chamber-associated immune deviation (ACAID). III. Induction of ACAID depends upon intraocular transforming growth factor-beta. Eur J Immunol.

[B50] Wilbanks GA, Streilein JW (1992). Fluids from immune privileged sites endow macrophages with the capacity to induce antigen-specific immune deviation via a mechanism involving transforming growth factor-beta. Eur J Immunol.

[B51] Takeuchi M, Alard P, Streilein JW (1998). TGF-beta promotes immune deviation by altering accessory signals of antigen-presenting cells. J Immunol.

[B52] Masli S, De Fazio SR, Gozzo JJ (2000). Requirement for early donor cell chimerism during prolonged survival of murine skin allografts. Transplantation.

[B53] Masli S, Turpie B, Streilein JW (2006). Thrombospondin orchestrates the tolerance-promoting properties of TGFbeta-treated antigen-presenting cells. International immunology.

[B54] Jakobs TC, Libby RT, Ben Y, John SWM, Masland RH (2005). Retinal ganglion cell degeneration is topological but not cell type specific in DBA/2J mice. J Cell Biol.

[B55] Libby RT, Li Y, Savinova OV, Barter J, Smith RS, Nickells RW, John SWM (2005). Susceptibility to neurodegeneration in a glaucoma is modified by Bax gene dosage. PLoS Genet.

[B56] Zdarsky E, Favor J, Jackson IJ (1990). The molecular basis of brown, an old mouse mutation, and of an induced revertant to wild type. Genetics.

[B57] Dell'Angelica EC, Mullins C, Caplan S, Bonifacino JS (2000). Lysosome-related organelles. Faseb J.

[B58] Raposo G, Fevrier B, Stoorvogel W, Marks MS (2002). Lysosome-related organelles: a view from immunity and pigmentation. Cell Struct Funct.

[B59] Li W, Rusiniak ME, Chintala S, Gautam R, Novak EK, Swank RT (2004). Hermansky-Pudlak syndrome genes: regulators of lysosome-related organelles. Bioessays.

[B60] Wei ML (2006). Hermansky-Pudlak syndrome: a disease of protein trafficking and organelle function. Pigment Cell Res.

[B61] John SW, Hagaman JR, MacTaggart TE, Peng L, Smithes O (1997). Intraocular pressure in inbred mouse strains. Invest Ophthalmol Vis Sci.

[B62] Savinova OV, Sugiyama F, Martin JE, Tomarev SI, Paigen BJ, Smith RS, John SWM (2001). Intraocular pressure in genetically distinct mice: an update and strain survey. BMC Genet.

[B63] Kontanis EJ, Reed FA (2006). Evaluation of real-time PCR amplification efficiencies to detect PCR inhibitors. J Forensic Sci.

[B64] Pfaffl MW (2001). A new mathematical model for relative quantification in real-time RT-PCR. Nucleic Acids Res.

[B65] Ramakers C, Ruijter JM, Deprez RH, Moorman AF (2003). Assumption-free analysis of quantitative real-time polymerase chain reaction (PCR) data. Neurosci Lett.

[B66] Mombaerts P, Iacomini J, Johnson RS, Herrup K, Tonegawa S, Papaioannou VE (1992). RAG-1-deficient mice have no mature B and T lymphocytes. Cell.

[B67] Blunt T, Gell D, Fox M, Taccioli GE, Lehmann AR, Jackson SP, Jeggo PA (1996). Identification of a nonsense mutation in the carboxyl-terminal region of DNA-dependent protein kinase catalytic subunit in the scid mouse. Proc Natl Acad Sci USA.

