# Supplementary figures and images for: GpnmbR150X allele must be present in bone marrow derived cells to mediate DBA/2J glaucoma
Source: BMC Genet. 2008 Apr 10;9:30. doi: 10.1186/1471-2156-9-30 (PMC2373794; doi:10.1186/1471-2156-9-30)

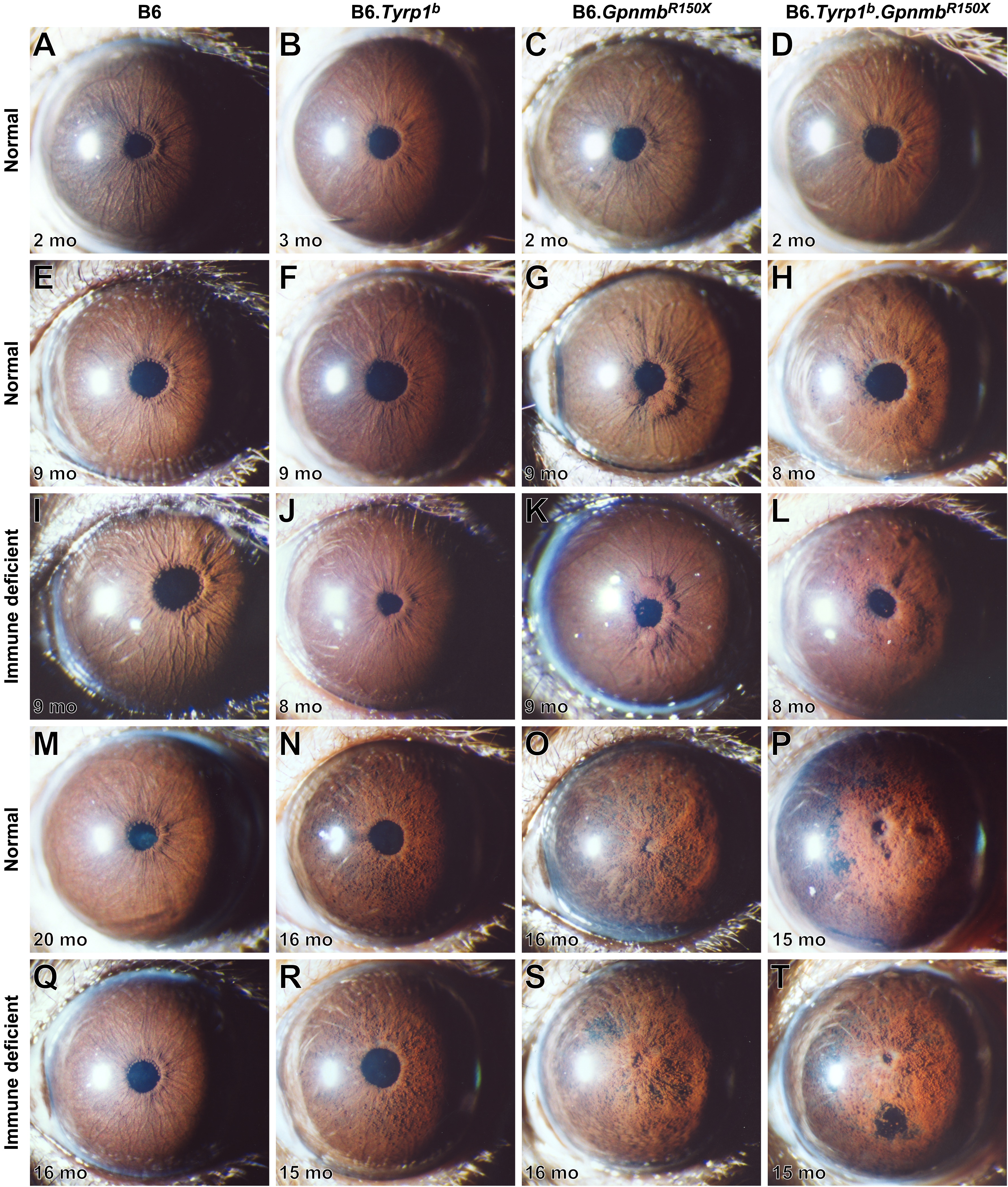

Supplement: Additional file 1 — Loss of adaptive immune reactions has no influence on the age of onset, rate of progression, or final severity of the Tyrp1 or Gpnmb mediated iris disease. The Rag1tm1Mom and Prkdcscid mutations both act recessively and result in essentially identical phenotypes characterized by loss of adaptive immune responses due loss of mature B and T lymphocytes. Stocks carrying each of these mutations were separately bred to B6.Tyrp1bGpnmbR150X mice. During the course of establishing triple mutant stocks, single and double mutant progeny classes were also isolated. Subsets of these were aged, with typical eyes of indicated genotypes shown. The combined analysis strikingly demonstrates that the immune deficiency associated with Rag1 or Prkdc mutation has no effect on Tyrp1 or Gpnmb mediated iris disease. Each column corresponds to a particular genotype with respect to Tyrp1 and Gpnmb; the rows are organized according to Rag1 or Prkdc genotype and age (homozygous mutants labeled as "Immune Deficient"; heterozygous and wild-type mice labeled "Normal"). (A to D) Eyes of young mice of all genotypes are characterized by healthy irides. By 8–9 mo, Tyrp1 and Gpnmb mutant mice exhibited typical signs of early disease that did not differ whether the mice were (E to H) immune competent, or (I to L) immune deficient. At 14+ mo, Tyrp1 and Gpnmb mutant mice exhibited signs of late disease that did not differ whether the mice were (M to P) immune competent or (Q to T) immune deficient. Panels K and Q are based on observations of 2 eyes, panel I from 6 eyes, and all other panels from groups of 8 to 26 eyes. [file 1471-2156-9-30-S1.jpeg]

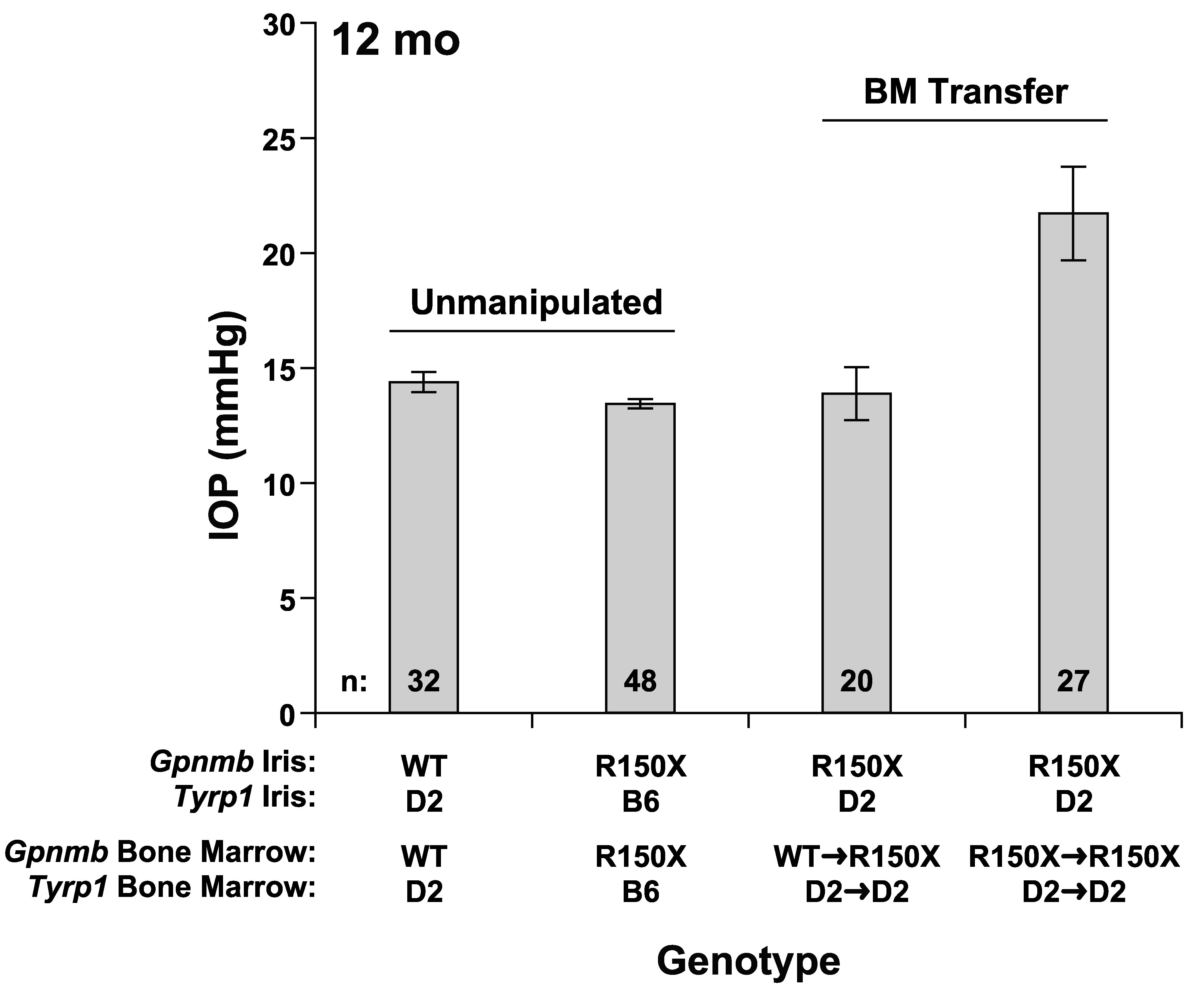

Supplement: Additional file 2 — GpnmbR150X mutation does not directly elevate IOP. Mean IOP ± SEM is presented. For ease of comparison, some of the data are duplicated from Figure 3. It is clear that the GpnmbR150X mutation contributes to the iris disease of DBA/2J mice and is a critical component of the glaucoma. Manipulations that prevent severe iris disease in DBA/2J mice prevent both anterior chamber enlargement (a marker of IOP elevation) and glaucoma development [20,22]. Thus, the iris disease is an essential component of the glaucoma. To determine if the GpnmbR150X mutation also influences IOP independently of the iris disease, we have assessed IOP in aged DBA/2J mice that have the Gpnmb mutation in all tissues but that do not develop the severe iris disease. These mice were homozygous for the wild-type C57BL/6J allele of Tyrp1 (Tyrp1B6, that was backcrossed into DBA/2J for more than 10 generations) [34] and the mutant DBA/2J allele of Gpnmb (GpnmbD2 = GpnmbR150X). Since mutant DBA/2J alleles of both Tyrp1 and Gpnmb are necessary to develop severe iris disease, the Tyrp1B6GpnmbD2 mice did not do so. Importantly and despite the presence of the Gpnmb mutation in the iris and bone marrow, the Tyrp1B6 GpnmbD2 mice did not develop elevated IOP. Thus, the GpnmbR150X mutation does not induce high IOP directly but does so by inducing the iris disease. [file 1471-2156-9-30-S2.jpeg]
